# A roller-coaster ride: Introduction of pentavalent vaccine in India

**Published:** 2011-06

**Authors:** Harish Nair, Indrajit Hazarika, Ashok Patwari

**Affiliations:** 1 Centre for Population Health Sciences and Global Health Academy, University of Edinburgh, Scotland, UK; 2 Public Health Foundation of India, New Delhi, India; 3 Center for Global Health and Development, Boston University, USA

Introduction of *Haemophilus influenzae* type B (HiB) containing pentavalent vaccines (a combination vaccine which protects against five killer diseases- diphtheria, pertussis, tetanus, hepatitis B and *Haemophilus influenzae* type B) in the Universal Immunization Program (UIP) was a far sighted decision taken in 2009 by the Ministry of Health and Family Welfare, Government of India. This decision was based on the recommendations of National Technical Advisory Group on Immunization (NTAGI) and was aimed at reducing the burden of HiB related infections (1). The decision was supported by the GAVI Alliance (formerly known as the Global Alliance for Vaccines and Immunizations) and in August 2009 they decided to provide funding worth US$ 165 million to the Government of India to support the introduction of pentavalent vaccine (2). The vaccine was to be introduced in a phased manner. In the first phase, the vaccine would have been rolled-out in 10 states and an estimated 18 million infants were expected to receive the vaccine. The decision of the Indian Government to introduce HiB vaccination into its UIP was hailed internationally by public health practitioners as India constitutes 34% of the birth cohort in GAVI-eligible countries (3) and even in the absence of population-based data, the country is estimated to have the highest number of deaths due to HiB in children under 5 years of age (4).

However, the plans to introduce the vaccine were stalled following the filing of a Public Interest Litigation (PIL) in the Delhi High Court in December 2010, which questioned rationale for introducing the vaccine as well as its efficacy (5). The petitioners, comprising of a mixed group of medical practitioners including paediatricians, policy advisors to the Government of India, and a former civil servant (who also oppose the introduction of Hepatitis B vaccine into the UIP) claimed inter alia that the NTAGI had based its recommendation without considering data from studies which reveal that the burden of meningitis caused by HiB in Indian children is much lower than in other parts of the world (6,7). Moreover, the petitioners claimed that recent evidence from countries which have used pentavalent vaccine for several years revealed that there was no real benefit to children (8). They also claimed that the vaccine had been withdrawn from neighbouring Bhutan and Sri Lanka after reports of adverse effects following immunization with the vaccine. The Delhi High court sought a reply from the Indian Council of Medical Research (ICMR), NTAGI and Indian Ministry of Health (9). Under increasing pressure, the government decided to halt the introduction of the vaccine and set up an expert committee to review all the available evidence on the HiB disease burden, assess the need for introducing pentavalent vaccine as a part of UIP and review the possible adverse effects. Although the findings of the expert committee have not been made public, recent reports in the media indicate that the Indian Government plans to introduce the vaccine in two South Indian states (Tamil Nadu and Kerala) in September 2011 (10,11). Amidst all this controversy, it is justified to question if the objections raised by the petitioners were based on sound evidence.

In the absence of surveillance data or good quality community based studies with active ascertainment of cases of invasive HiB disease to base the burden of disease estimate in India, the only option is to use data derived from hospital – based studies with passive case ascertainment and mathematical models based on systematic literature review and vaccine probe studies. Using the latter approach, Watt and colleagues estimated the burden of invasive HiB disease (which includes pneumonia and meningitis) in India in 2000 to be about 2.4 million cases with 72 000 deaths in children aged less than 5 years, which accounted for approximately 4% of all child deaths in India (4). It is well acknowledged that the incidence of pneumonia far exceeds meningitis while the latter has a higher case fatality ratio. In 2008, Rudan and colleagues estimated that in India, 43 million new cases of clinical pneumonia in children under the age of 5 years occur each year and result in 408 000 deaths (12). Using the estimates from the HiB study (4), we estimate that around 215 000 new cases of HiB pneumonia occur yearly in Indian children under the age of 5 years and result in over 61 000 deaths. Thus, studies have consistently projected the burden of HiB disease in India to be significant. The studies cited by the petitioners (7,13) have serious limitations, making it extremely difficult to generalise the results to the Indian population as a whole – the studies were conducted more than 15 years ago in an area which had, even at that time, less than half the infant mortality rate compared to the Indian average; the investigators only looked at HiB meningitis as an outcome; and have themselves concluded that “these estimates are minimal” (7). However, in the light of the present controversy, it may be worthwhile to conduct a systematic literature review of Indian studies to estimate the burden of HiB related acute bacterial pneumonia and meningitis in India to provide a clearer picture.

The concerns raised regarding the adverse effects of the pentavalent vaccine appear to be unsubstantiated. The World Health Organization had established a panel of international experts to examine the reports of hypotonic hyporesponsive episodes (HHEs) following administration of the pentavalent vaccine (HHE is a recognized adverse reaction to whole-cell and acellular pertussis-containing vaccines, and to HiB and hepatitis B vaccines). The expert panel concluded that there was no evidence to establish a causal relationship between pentavalent vaccine and any of the deaths reported following its administration (14). It also concluded that “the reporting rate of HHE following the pentavalent vaccine (14.9 cases per 100 000 doses) was found to be well within the reported estimates of HHE following whole-cell pertussis-containing vaccines (21–250 cases per 100 000 doses).” Following this, the Sri Lankan government decided to re-introduce the vaccine from September 2009. However, due to shortage of fresh stocks this was only possible in February 2010 (15).

Contrary to the remarks on the lack of beneficial effects of the vaccine, 150 countries across the globe that have already introduced HiB vaccine have reported a dramatic decline in the incidence of invasive HiB disease and death. Morris and colleagues in a systematic review of the effectiveness of HiB vaccine demonstrated that HiB conjugate vaccines were highly effective in reducing the incidence of invasive HiB disease, with similar effectiveness seen across geographical regions and different levels of socioeconomic development. (16) Even in countries which have poor immunization coverage, indirect benefits of the vaccine have been reported due to the herd effect. For instance, data from Gambia have shown the benefits of herd immunity even when vaccine coverage has been below 60% (17). The vaccine should thus be effective in India where UIP coverage is poor. In fact, long before the NTAGI recommended introduction of HiB vaccine into the UIP, the Indian Academy of Pediatrics had called for incorporating the vaccine into the UIP (18).

The Cochrane study which has been cited by the petitioners to show that there is no benefit of a combination vaccine in terms of disease burden reduction and immunogenicity found that no studies reported the primary outcome for the study ie, incidence of disease (8). The authors themselves conclude that “[T]he results of this review should be viewed with caution, mostly as an indication that high quality data are lacking.” Moreover, Dutta and colleagues recently carried out a phase-III multicentric trial of the pentavalent vaccine and found that the combination vaccine had a high immunogenicity and was well tolerated (19). In resource-poor settings like India, decisions to use the vaccine are expected to be guided by the cost associated with its introduction. While the concerns regarding the costs are legitimate, recent data suggest that the cost of the vaccine has reduced substantially. At present there are at least five Indian companies manufacturing the vaccine. With one of the Indian manufacturers, the Serum Institute of India, announcing in June 2011 that they plan to sell the vaccine at US$ 1.75 (€ 1.2) per dose, it is expected that the other manufacturers will follow suit (20). In the future the price will reduce even further – as a result of bulk procurement by the Government and competition between the manufacturers. It has already been demonstrated that any price lower than US$ 2 (€ 1.4) per dose is highly cost-effective (21).

**Figure:**
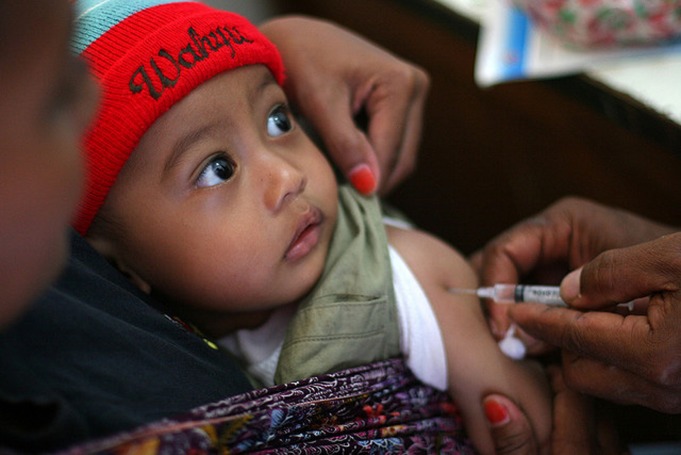
Photo: Courtesy of UNICEF Sverige

The concerns regarding the introduction of a new vaccine on an already overwhelmed public health system in India appear to be valid. However, experience from other developing countries suggests that it is feasible despite limited resources (22). Further, it may not be inappropriate to assume that the opportunity offered by the introduction of a new vaccine may provide the desired boost to the health system through refresher trainings to the health workers and generating demand among parents and caregivers and may lead to an improvement in the routine immunization coverage especially in the North Indian states. Hence, while the decision regarding GAVI Alliance’s efforts to introduce the pentavalent vaccine in India seems to be centred around debates regarding the associated commercial considerations, it may be prudent to focus more on the long-term benefits of the vaccine and its potential to reduce mortality and morbidity amongst children aged less than 5 years, bringing the country closer to Millennium Development Goal 4.
